# Crystal structure and mutagenesis of a nucleic acid–binding BRCT domain in human PARP4

**DOI:** 10.1016/j.jbc.2025.110277

**Published:** 2025-05-22

**Authors:** Léonie Frigon, John M. Pascal

**Affiliations:** Department of Biochemistry and Molecular Medicine, Université de Montréal, Montréal, Qc, Canada

**Keywords:** PARP enzyme, BRCT fold, vault RNA, X-ray crystallography, nucleic acid binding

## Abstract

PARP4 is an ADP-ribosyltransferase typically associated with the cytoplasmic vault organelle. PARP4 has a distinct domain composition relative to other PARP enzymes; however, the N-terminal region of PARP4 is homologous to a collection of domains found in PARP1, a regulator of multiple nuclear processes including the cellular response to DNA damage. The N-terminal region of PARP4 interacts *in vitro* with nucleic acid, in particular a noncoding RNA associated with vault particles, and a BRCT domain is implicated in this interaction. Here, we report the X-ray structure of the BRCT domain of PARP4 and structure-based mutagenesis that interrogates the nucleic acid–binding activity using vault RNA. The isolated BRCT domain is capable of mediating interaction with vault RNA, and we identified four BRCT mutants that disrupt vault RNA interaction to varying degrees. X-ray structures of the BRCT mutants indicate that perturbations to an electropositive region of the BRCT surface underlie the loss of nucleic acid binding. Comparison to other nucleic acid–binding BRCT domains highlights distinct features of the PARP4 BRCT structure. The study presents the experimental structure of the PARP4 BRCT domain, establishes this domain as nucleic acid–binding module, and provides PARP4 BRCT mutants that can be used to investigate PARP4 cellular functions.

PARP4 is an ADP-ribosyltransferase (ART) that uses NAD^+^ as substrate to add ADP-ribose onto target proteins, a process called mono(ADP-ribosylation) or MARylation (1). PARP4 is also referred to as vault PARP since it can localize to the vault particle, a cytoplasmic ribonucleoprotein complex (2, 3, 4). The human PARP family is composed of 17 canonical members that can be separated into 6 clades based on structural and evolutionary similarity, with PARP4 being the only human member of the fifth clade and having a unique domain composition (5). However, the BRCT-WGR-catalytic (CAT) collection of domains found at the C terminus of human PARP1 is homologous to the N terminus of PARP4 (Fig. 1*A*). The central part of PARP4 is predicted to contain an inter-alpha-trypsin domain and a von Willebrand factor type A domain and is referred to as the inter-alpha-trypsin inhibitor heavy chain–like region (Fig. 1*A*). The biochemical properties and structure of the inter-alpha-trypsin inhibitor heavy chain–like region are not known. A major vault protein interacting domain is found at the PARP4 C terminus. The major vault protein interacting domain is responsible for PARP4 localization to the vault organelle (2, 6). There is very little experimental structure information and biochemical analysis of domain activities for PARP4, limiting our understanding of PARP4 function, mechanism, and regulation.

**Figure 1:**
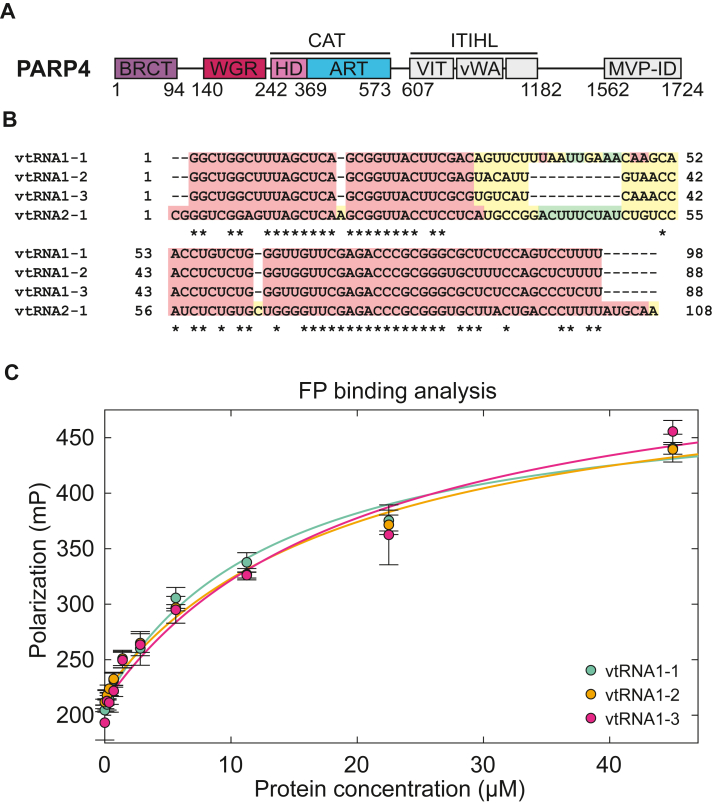
**PARP4 BRCT domain preferentially binds to vtRNA1-3.***A*, schematic representation of PARP4. *B*, sequence alignment of the four human vault RNA paralogs using the TCoffee server (43). *Red* indicates good sequence conservation, *yellow* is average conservation, and *green* is bad conservation. *C*, FP binding assay using 3 nM vtRNA (1–1, 1–2, or 1–3) bearing a fluorescent label. The BRCT domain was incubated with each vtRNA at the designated concentrations. The data points and error bars represent the average and SD of the independent experiments. A 1:1 binding model was fit to the binding curves, yielding an apparent K_D_ of 13 ± 4 μM for vtRNA1-1, 20 ± 5 μM for vtRNA1-2, and 19 ± 4 μM for vtRNA1-3 (Table 1). FP, fluorescence polarization; vtRNA, vault RNA. VIT, vault inter-alpha-trypsin domain; vWA, von Willebrand factor type A domain; ITIHL, inter-alpha-trypsin inhibitor heavy chain-like region; MVP-ID, major vault protein interacting domain.

The vault organelle is an enormous eukaryotic ribonucleoprotein organelle (∼13 MDa) for which the precise function is still a mystery (2, 3, 4). Vault organelles are upregulated in multidrug-resistant, non–P-glycoprotein cancer cell lines, hinting toward a role in drug resistance mechanisms (7). Vault is composed of 78 copies of major vault protein that assemble into two equivalent halves of a barrel-shaped organelle (8). Previous studies estimated that the vault contains two copies of telomerase-associated protein 1, eight copies of PARP4, and at least six copies of a noncoding RNA known as vault RNA (vtRNA) (9). There are four vtRNA paralogs and three vtRNA related genes in the human genome, but only three paralogs interact with the vault organelle: vtRNA1-1, vtRNA1-2 and vtRNA1-3 (10, 11). Human vtRNAs have similar predicted secondary structures with sequence identity between 64% and 70% and similar lengths between 88 and 108 nucleotides (Figs. 1*B* and S1). vtRNA that is associated with the vault organelle represents 5% of cellular vtRNA, and the remaining vtRNA is distributed in the cytoplasm and suggested to participate in processes such as cell proliferation, signaling pathways, apoptosis, and innate immune response (11). The functional role of vtRNA within the vault organelle is not known. Given the colocalization of vtRNA and PARP4 within the vault organelle, and the collection of PARP4 domains that are typically associated with nucleic acid interaction, we have explored the interaction between PARP4 and vtRNA as a first step toward understanding potential nucleic acid–binding functions of PARP4. Whether the PARP4 interaction with vtRNA is relevant to vault organelle function, or other vtRNA functions, remains to be determined.

In a previous study, we examined the structure and MARylation activity of the BRCT-WGR-CAT region of PARP4 that is homologous to PARP1 (12). The PARP4 CAT region is composed of a helical domain (HD) and an ART fold, similar to PARP1. The HD of PARP1 is potently autoinhibitory, preventing the PARP1 ART from binding substrate NAD^+^ (13, 14). HD autoinhibition is relieved when PARP1 domains, including the WGR, bind to a DNA break and alter the HD structure (13, 15, 16). In contrast, the HD of PARP4 appears to adopt a natively open conformation that allows NAD^+^ access to the ART and thus does not autoinhibit (12). The BRCT-WGR-CAT region of PARP4 binds to vtRNA with micromolar affinity, and this vtRNA binding activity is disrupted when the BRCT is removed; however, PARP4 MARylation activity does not appear to be impacted by binding to vtRNA (12). The PARP4 BRCT and WGR domains can regulate the MARylation activity of the PARP4 CAT, acting to downmodulate CAT output relative to the CAT domain alone (12), but the basis for this regulation is not clear. It is not known whether the isolated BRCT domain can mediate vtRNA binding activity.

The BRCT domain was identified in the C-terminal region of breast cancer–associated protein 1 and thus named breast cancer–associated protein 1 C terminus or BRCT (17). BRCT domains are involved in an array of activities such as phospho-peptide binding, phosphorylation-independent protein interactions, DNA binding, RNA binding, and poly(ADP-ribose) binding (17). BRCT domains are found in a variety of proteins, most of which are involved in the DNA damage response (18, 19). BRCT domains are found as single units, or in tandem assemblies of two or three BRCT domains, allowing them to have high versatility of specificity and regulation (17). This versatility also makes it difficult to predict the potential roles of the PARP4 BRCT domain.

In this study, we undertook a structural and biochemical analysis of the human PARP4 BRCT domain. We establish that the isolated BRCT domain can bind vtRNA. We determined a 1.75 Å crystal structure of the PARP4 BRCT domain that allowed us to compare its structure to other BRCT domains with nucleic acid–binding activity. From this structural analysis, we targeted residues deemed likely to contribute to nucleic acid binding. The mutants indeed showed moderate to severe phenotypes in vtRNA binding assays. We determined crystal structures of the BRCT mutants and observed that the mutations lead to perturbations to the electrostatic surface potential that likely underlie the deficiencies in binding. The most severe mutant was analyzed in the context of the BRCT-WGR-CAT region and exhibited a deficiency in vtRNA interaction equivalent to a BRCT deletion from this region. Analysis of BRCT-WGR-CAT and isolated BRCT interaction with fragments of vtRNA1-3, and vtRNA1-3 composed of DNA nucleotides, suggest a general mode of interaction with nucleic acid rather than a structure-specific interaction. Collectively, the study establishes the human PARP4 BRCT as a nucleic acid–binding module. The identification of a mutant that abolishes BRCT interaction with vtRNA will aid future studies that test the associated phenotype in cells and that could lead to a better understanding of PARP4 function.

## Results

### PARP4 BRCT binds to vtRNA

We previously showed that PARP4 BRCT-WGR-CAT fragment binds vtRNA1-2 with micromolar affinity (12). Under these assay conditions, we showed that a deletion of the BRCT domain largely abolished the interaction; however, we did not test whether the BRCT alone could mediate the interaction with vtRNA. We thus performed fluorescence polarization (FP) binding analysis using chemically synthesized and fluorescently labeled vtRNAs to assess the capacity of the isolated BRCT domain to bind nucleic acid. We used the three vtRNAs known to interact with the vault particle: vtRNA1-1, vtRNA1-2, and vtRNA1-3 (10). Indeed, the BRCT exhibited similar binding to each of the vtRNA (Fig. 1*C*), indicating that the BRCT domain alone is capable of mediating nucleic acid interaction. The apparent equilibrium binding constant (K_D_) was similar within the margin of error and ranged from 13 to 20 μM (Table 1). There is some uncertainty in the apparent K_D_ values from the FP assay due to limitations in protein quantity that prevented a robust determination of fully saturated binding, which is more evident when the binding data is plotted *versus* the log of protein concentration (Fig. S2*A*). Despite this limitation, the FP binding analysis provides a suitable evaluation of relative affinities, and we also use EMSA analysis to support our key findings.

**Table 1:** Equilibrium binding constants determined by FP and EMSA

PARP4 fragment	Ligand	FP (μM)^a^	EMSA (μM)^a^
BRCT	vtRNA1-3	19 ± 4	0.5 ± 0.2
BRCT	vtRNA1-1	13 ± 4	Not measured
BRCT	vtRNA1-2	20 ± 5	Not measured
BRCT-WGR-CAT	vtRNA1-3	27 ± 9	3 ± 1
BRCT	vtDNA1-3	10 ± 3	0.5 ± 0.2
BRCT F39Q	vtRNA1-3	35 ± 10	0.9 ± 0.5
BRCT F39A	vtRNA1-3	33 ± 2	0.6 ± 0.1
BRCT K31Q	vtRNA1-3	61 ± 12	3 ± 1
BRCT K23/24Q	vtRNA1-3	No binding	No binding
BRCT-WGR-CAT K23/24Q	vtRNA1-3	71 ± 26	54 ± 9
WGR-CAT	vtRNA1-3	93 ± 23	No binding
BRCT-WGR-CAT	Probe 1	25 ± 10	11 ± 3
BRCT-WGR-CAT	Probe 2	44 ± 13	11 ± 3
BRCT-WGR-CAT	Probe 3	No binding	11 ± 2
BRCT-WGR-CAT	Probe 4	No binding	7 ± 2

CAT, catalytic; FP, fluorescence polarization; vtRNA, vault RNA.

a Average K_D_ values and SDs obtained from three independent biological replicates (n = 3), except for FP with BRCT WT and vtRNA1-3 (n = 9), FP with BRCT-WGR-CAT and vtRNA1-3 (n = 10), EMSA with BRCT WT and vtRNA1-3 (n = 7), and EMSA with BRCT-WGR-CAT and vtRNA1-3 (n = 12). No binding: no apparent interaction.

**Figure 2:**
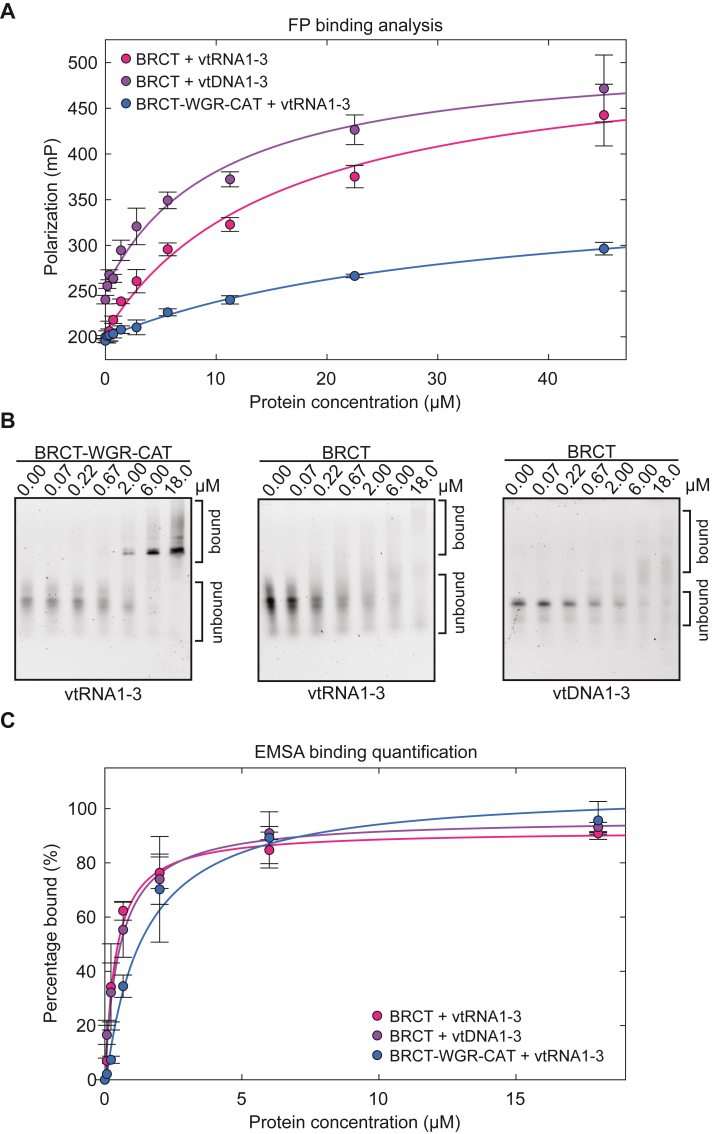
**PARP4 BRCT domain equally binds to vtRNA1-3 and vtDNA1-3.***A*, FP binding assay using 3 nM of vtRNA1-3 or vtDNA1-3 bearing a fluorescent label. PARP4 fragments (BRCT or BRCT-WGR-CAT) were incubated with the designated nucleic acid at the different protein concentrations. The data point and error bars represent the average and SD of three independent experiments. A 1:1 binding model was fit to the binding curves. Binding of the BRCT domain to vtDNA1-3 yielded an apparent K_D_ of 10 ± 3 μM. An apparent K_D_ of 19 ± 4 μM was obtained for the binding of the BRCT domain to vtRNA1-3. BRCT-WGR-CAT fragment binds to vtRNA1-3 with an apparent K_D_ of 27 ± 9 μM. *B*, EMSA using 20 nM vtRNA1-3 bearing a fluorescent label. BRCT-WGR-CAT or BRCT fragment was incubated with vtRNA1-3 or vtDNA1-3 at the indicated concentrations. These are representative experiments that have been repeated at least three times each (see Table 1). *C*, quantification of the repeats of the EMSA experiments from *panel (B)*. The data points and error bars represent the average and SD of the independent experiments. A 1:1 binding model was fit to each binding curve. Binding of the BRCT-WGR-CAT fragment to vtRNA1-3 yielded an apparent K_D_ of 3 ± 1 μM. Binding of the BRCT domain to vtRNA1-3 yielded an apparent K_D_ of 0.5 ± 0.2 μM. A K_D_ of 0.5 ± 0.2 μM was obtained from the binding of the BRCT domain to vtDNA1-3. CAT, catalytic; vtRNA, vault RNA.

Using the FP binding assay and EMSA analysis, we directly compared BRCT-WGR-CAT and BRCT binding to vtRNA1-3 (Fig. 2*A*). The FP experiment with BRCT-WGR-CAT exhibited an apparent K_D_ of 27 ± 9 μM, indicating a less robust affinity than the interaction with the BRCT alone with a K_D_ of 19 ± 4 μM (Fig. 2*A* and Table 1 and Fig. S2*B*). BRCT alone reached a higher level of polarization relative to BRCT-WGR-CAT, despite having a smaller mass, potentially indicating that more than one BRCT molecule binds to vtRNA1-3. Using EMSA analysis, BRCT-WGR-CAT exhibited a fairly well-defined band for the shift in bound probe, whereas BRCT produced a more diffuse signal (Fig. 2*B*), perhaps also reflecting that multiple BRCT domains are interacting with the probe. Due to the diffuse signal, the disappearance of unbound probe was used to quantify the EMSA binding data for the BRCT domain. The BRCT-WGR-CAT fragment and vtRNA1-3 yielded an apparent K_D_ of 3 ± 1 μM, and the isolated BRCT exhibited an apparent K_D_ of 0.5 ± 0.2 μM (Fig. 2*C* and Table 1 and Fig. S2*C*). As observed previously with BRCT-WGR-CAT binding to vtRNA1-2 (12), the FP binding assay and EMSA analysis yielded different estimates of binding affinities. The difference in K_D_ values between EMSA and FP experiments is likely explained by the different physical principles and measurement signals of the two assays. For EMSA, the intensity of the multiple shifted bands is collectively quantified. For FP, the dominant measurable signal might only emerge from a probe bound to multiple proteins. This species would correspond to the slower migrating bands on EMSA, which are only observed at elevated protein concentrations. The consequence is higher K_D_ values from FP experiments relative to the EMSA-derived K_D_ values, and this interpretation indicates that FP experiments are likely to have underestimated affinities. As noted above, we were not able to achieve a binding plateau in the FP assay due to the high concentrations required, thus making the FP-derived K_D_ values less reliable. However, the FP assay still provides a valid assessment of relative binding capacities so we continue to use this assay in conjunction with EMSA analysis. Both FP and EMSA binding experiments indicated a higher binding affinity for the isolated BRCT domain relative to BRCT domain in the context of WGR and CAT domains. This observation suggests that the BRCT domain is the main nucleic acid–binding module of the BRCT-WGR-CAT fragment and suggests that the WGR and CAT domains might interfere or interplay with the binding capacity of the BRCT domain.

Our previous work indicated a strong preference for BRCT-WGR-CAT to interact with vtRNA relative to ssDNA. However, there was not a strong preference for vtRNA relative to duplex DNA, thus suggesting that the BRCT-WGR-CAT region exhibits a general interaction with duplex nucleic acid structures (12). To assess the specificity of the isolated BRCT for RNA *versus* DNA, we used FP binding and EMSA to compare the interaction with both types of nucleic acids. The vtRNA1-3 sequence was converted to DNA to create vtDNA1-3, which was chemically synthesized and fluorescently labeled. Both nucleic acids thus have the same number of nucleotides and generally have the same potential to form duplex regions. Any differences in interaction would suggest specificity toward one type of nucleic acid. Using EMSA, the interaction of PARP4 BRCT with vtDNA1-3 yielded an apparent K_D_ of 0.5 ± 0.2 μM, which is essentially identical to the interaction of the BRCT with vtRNA1-3 (Fig. 2, *B* and *C* and Table 1 and Fig. S2*C*). Using an FP binding assay, we measured an apparent K_D_ of 10 ± 3 μM for vtDNA1-3 (Fig. 2*A* and Table 1 and Fig. S2*B*), compared to a K_D_ of 19 ± 4 μM for vtRNA1-3. The results are consistent with the BRCT region exhibiting general binding to nucleic acids, with no clear preference for RNA or DNA, at least in the vtRNA sequence and structure context.

### Analysis of BRCT-WGR-CAT binding to vtRNA1-3 fragments

**Figure 3:**
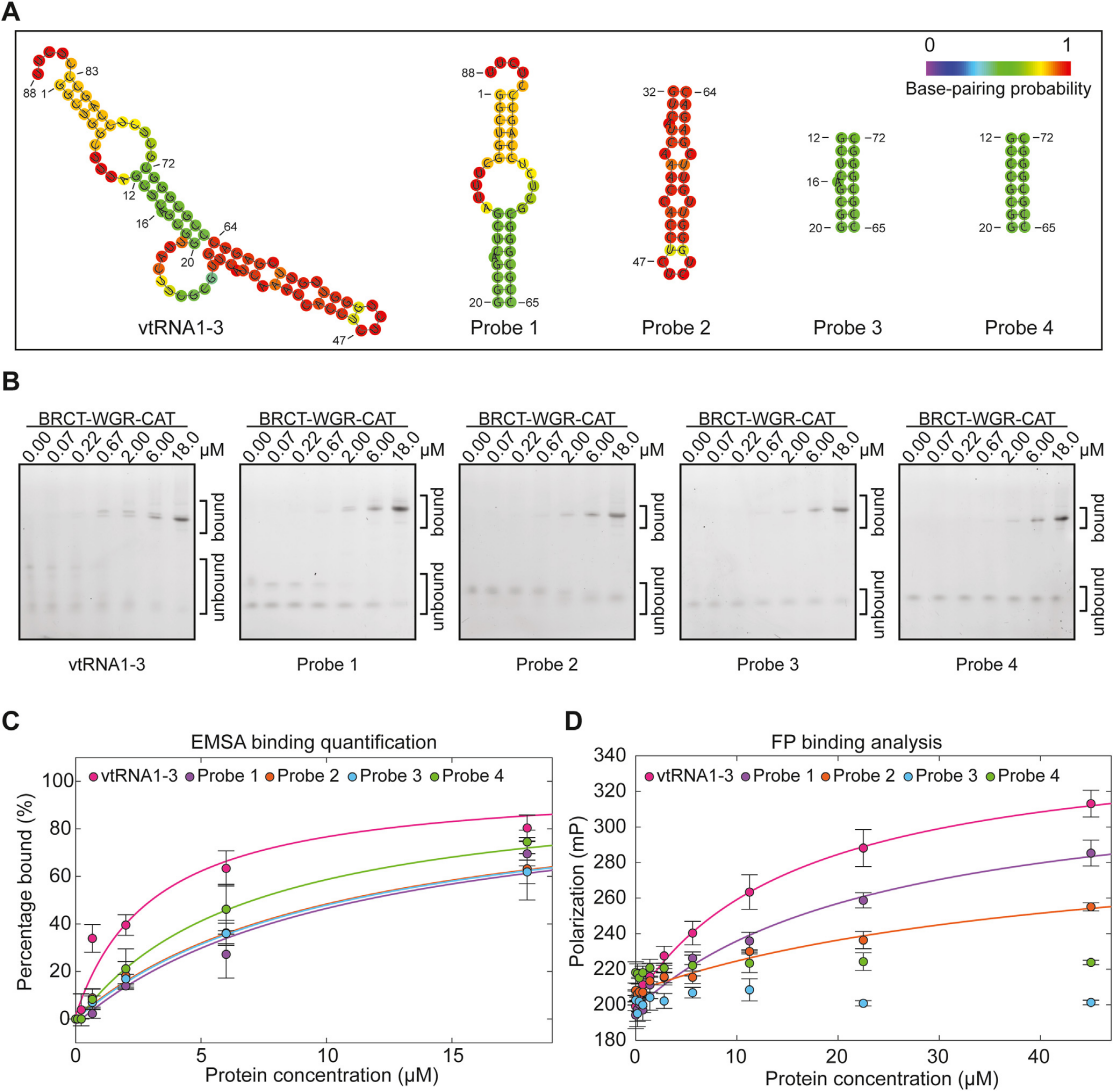
**PARP4 BRCT-WGR-CAT interaction with vtRNA structural elements.***A*, secondary structure prediction of vtRNA1-3 using RNA fold and the isolated regions used as probes (44). *B*, EMSA using 20 nM vtRNA1-3 or probe 1 to 4 bearing a fluorescent label. BRCT-WGR-CAT fragment was incubated with the nucleic acid at the indicated concentrations. These are representative experiments that have been repeated at least three times each (see Table 1). *C*, quantification of the independent biological repeats of the EMSA experiments from *panel (B)*. The data points and error bars represent the average and SD of the independent experiments. A 1:1 binding model was fit to each binding curve. Binding of the BRCT-WGR-CAT fragment to vtRNA1-3 yielded an apparent K_D_ of 3 ± 1 μM. An apparent K_D_ of 11 ± 3 μM was obtained for probe 1 and 3. An apparent K_D_ of 11 ± 2 μM was obtained for probe 3 and 7 ± 2 μM for probe 4. *D*, FP binding assay using 15 nM of vtRNA1-3 or probe 1 to 4 bearing a fluorescent label. PARP4 BRCT-WGR-CAT was incubated with the designated nucleic acid at the different protein concentrations. The data points and error bars represent the average and SD of three independent experiments. A 1:1 binding model was fit to the binding curves. Binding of BRCT-WGR-CAT to vtRNA1-3 yielded an apparent K_D_ of 27 ± 9 μM. An apparent K_D_ of 25 ± 10 μM and 44 ± 13 μM were determined for probe 1 and 2, respectively. It was not possible to determine a K_D_ value for the binding of BRCT-WGR-CAT to probe 3 and 4. CAT, catalytic; vtRNA, vault RNA; FP, fluorescence polarization.

We further investigated BRCT-WGR-CAT interaction with nucleic acid by analyzing the different structural elements present in vtRNA1-3. Based on the predicted secondary structure, vtRNA1-3 is largely composed of duplex RNA with some internal loops and bulges and unpaired regions (Fig. 3*A*). We created four probes that were designed to isolate different secondary structure elements (Fig. 3*A* and Table S1 for sequences). Probe 1 represents roughly one-half of the vtRNA1-3 structure, and probe 2 essentially represents the other half. Probe 3 represents a central duplex region with a single unpaired A nucleotide, and in probe 4 the unpaired A nucleotide is not present yielding pure duplex. Using EMSA analysis, we observed the following relative K_D_ values: 11 ± 3 μM for probe 1 and probe 2, 11 ± 2 μM for probe 3, and 7 ± 2 μM for probe 4 (Fig. 3, *B* and *C* and Table 1 and Fig. S3*A*). The K_D_ value of 3 ± 1 μM was measured for the complete vtRNA1-3, thus each of the fragments exhibited weakened interaction. FP analysis of the same probe set yielded the following estimates for K_D_ values: 25 ± 10 μM for probe 1 and 44 ± 13 μM for probe 2 (compared to 27 ± 9 μM for complete vtRNA1-3). Probes 3 and 4 did not show strong evidence of interaction and K_D_ values were not estimated (Fig. 3*D* and Table 1 and Fig. S3*B*). The lack of apparent binding in the FP analysis using the smaller probes 3 and 4 supports the idea that the measurable FP signal largely originates from a multiply bound species that is not possible on a smaller probe. Taken together, the results of this binding analysis indicate that BRCT-WGR-CAT does not have preferential binding to a specific structural element of vtRNA1-3.

### PARP4 BRCT crystal structure

**Figure 4:**
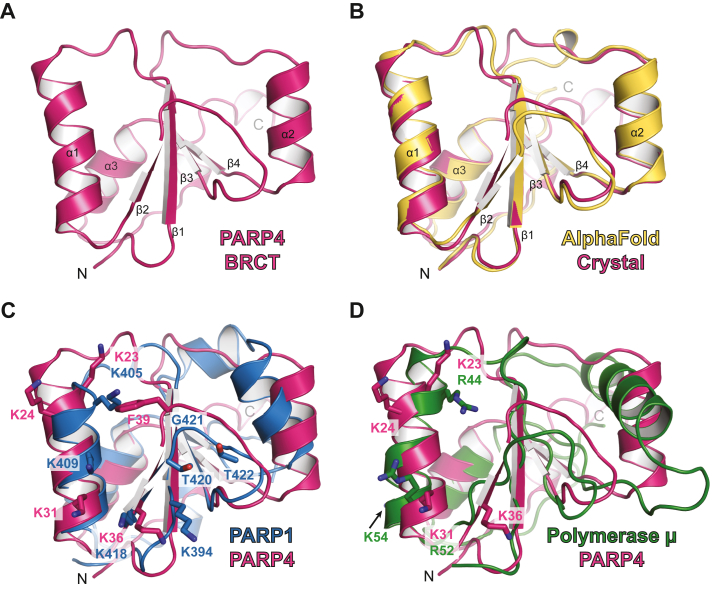
**Crystal structure of PARP4 BRCT domain.***A*, crystal structure of PARP4 BRCT domain at a resolution of 1.75 Å (PDB: 9DEV; from this work). The canonical alpha-helices and beta-strands from the BRCT fold are labeled. *B*, comparison of the crystal structure of PARP4 BRCT domain (drawn in *pink*) to the AlphaFold predicted structure (drawn in *yellow*). Superimposition of the structures yielded a RMSD of 2.0 Å (comparing 751 atoms). *C*, comparison of PARP4 BRCT domain (drawn in *pink*) to the PARP1 BRCT domain (drawn in *blue*, PDB: 7SCY). Superimposition of the structures yielded a RMSD of 3.0 Å (comparing 81 Cα atoms). Key residues for DNA binding are labeled on the PARP1 structure and their corresponding PARP4 residues or other potential key residues are also labeled. *D*, comparison of the PARP4 BRCT domain (drawn in *pink*) to polymerase μ (Polμ) (drawn in *green*, PDB: 2HTF). Superimposition of the structures yielded a RMSD of 4.3 Å (comparing 74 Cα atoms). Key residues for Polμ binding to dsDNA and their corresponding residues in PARP4 are labeled. vtRNA, vault RNA.

We pursued X-ray crystallography to obtain a detailed understanding of the PARP4 BRCT domain structure and the features that could contribute to nucleic acid binding. The BRCT domain was crystallized using sitting drop vapor diffusion. Molecular replacement phasing of the diffraction data was realized using the AlphaFold predicted structure of PARP4 BRCT domain (20, 21). Crystals of the BRCT domain diffracted to 1.75 Å and belong to space group F23 with one molecule in the asymmetric unit; the refined structure has a crystallographic R/R_FREE_ of 0.175/0.188 (Table 2). As expected, the PARP4 BRCT domain adopts the characteristic fold seen in other BRCT domains, with a central four-stranded β-sheet (β1-4) flanked by two α-helices (α1 and α3) on one side of the sheet and a single α-helix (α2) on the other side (Fig. 4*A*; PDB: 9DEV) (17). The AlphaFold predicted model was a successful search model for molecular replacement, providing phases that yielded a readily interpretable starting electron density map. However, there were several regions that required significant manual adjustments, and the final refined X-ray structure relative to the starting AlphaFold structure yielded an RMSD of 2.0 Å (comparing all 751 main chain and sidechain atoms) (Fig. 4*B*). The major differences exist in loop regions connecting the core secondary structure elements, for example, the residues connecting strands β2 and β3 in the central beta-sheet. As shown below, this region contributes to the nucleic acid–binding surface.

**Table 2:** Crystallographic statistics

Data Collection^a^					
Structure	**BRCT WT**	**BRCT K23/24Q**	**BRCT K31Q**	**BRCT F39A**	**BRCT F39Q**
PDB ID	9DEV	9DFP	9DFO	9DFR	9DFQ
Space group	F 2 3	F 2 3	F 2 3	F 2 3	F 2 3
Unit cell dimensions	a = b = c = 114.4 Å, α = β = γ = 90°	a = b = c = 114.7 Å, α = β = γ = 90°	a = b = c = 114.8 Å, α = β = γ = 90°	a = b = c = 115.4 Å, α = β = γ = 90°	a = b = c = 115.2 Å, α = β = γ = 90°
Wavelength (Å)	1.12	1.18	1.18	1.18	1.18
Resolution range (Å)	20–1.75 (1.78–1.75)	20–1.92 (1.97–1.92)	20–1.90 (1.94–1.90)	20–1.90 (1.95–1.90)	20–2.10 (2.16–2.10)
Completeness (%)	99.9 (100)	99.9 (100)	99.9 (99.9)	99.9 (100)	99.9 (100)
Unique observations	12,637 (714)	9666 (656)	9972 (653)	10,148 (687)	7499 (610)
Average redundancy	8.7 (8.3)	8.7 (8.5)	8.6 (8.1)	8.6 (8.4)	8.8 (9.0)
Mean (I/σI)^b^	23.7 (1.9)	23.7 (1.9)	36.9 (4.0)	22.3 (2.1)	32.1 (2.9)
R_merge_ (%)^b^	4.2 (85.1)	5.5 (105.4)	3.5 (61.6)	5.9 (101.6)	4.5 (74.2)
R_pim_ (%)^b^	1.5 (31.2)	2.0 (38.2)	1.3 (23.0)	2.1 (37.3)	1.6 (26.2)
Mean I CC (1/2)^b^	0.999 (0.775)	1.000 (0.621)	1.000 (0.837)	0.999 (0.607)	1.000 (0.815)
Model refinement^a^					
Resolution range (Å)	20–1.75 (1.80–1.75)	20–1.92 (1.97–1.92)	20–1.90 (1.95–1.90)	20–1.90 (1.95–1.90)	20–2.10 (2.16–2.10)
Number of reflections	11,958 (865)	9185 (660)	9453 (716)	9612 (695)	7112 (521)
R^c^	0.175 (0.286)	0.175 (0.263)	0.170 (0.238)	0.182 (0.284)	0.181 (0.260)
R_free_^c^	0.188 (0.328)	0.219 (0.358)	0.208 (0.285)	0.218 (0.378)	0.230 (0.290)
Number of atoms/average B-factor (Å^2^)					
Protein	832/45.2	801/41.5	800/38.8	796/40.4	809/50.9
Solvent	86/52.5	103/51.4	138/50.4	115/50.5	78/55.8
Phi/Psi, preferred (%)/outliers (#)^d^	96.47/0	97.659/0	97.62/0	97.50/0	97.59/0
RMSD bond angles (°)	1.276	1.210	1.259	1.257	1.283
RMSD bond lengths (Å)	0.004	0.004	0.004	0.004	0.004

a Values in parentheses refer to data in the highest resolution shell.

b As calculated in AIMLESS (34): R_merge_ = ∑_hkl_∑_j_∣I_j_ – ⟨I⟩∣/∑_hkl_∑_j_ I_j_. ⟨I⟩ is the mean intensity of *j* observations of reflection *hkl* and its symmetry equivalents; R_pim_ takes into account measurement redundancy when calculating R_merge_; mean I CC (1/2) is the correlation between mean intensities calculated for two randomly chosen half-sets of the data.

c R = ∑_hkl_∣F_obs_ – *k*F_calc_∣/∑_hkl_∣F_obs_∣ for reflections used in refinement. R_free_ = R for 5% of reflections excluded from crystallographic refinement.

d As reported in COOT (38).

With the high-resolution structure of the PARP4 BRCT, we identified regions that could be targeted for mutagenesis to verify an authentic interaction with nucleic acid, as opposed to a nonspecific interaction. Candidate regions were identified by examining the electrostatic surface potential for areas of concentrated positive charge that might engage the negatively charged backbone of nucleic acid. We also compared the PARP4 BRCT domain with other BRCT domain structures known to engage nucleic acid. We compared our PARP4 BRCT structure (PDB: 9DEV) with the PARP1 BRCT structure bound to nucleosomal DNA (PDB: 7SCY) (22). The superimposition of both BRCT domains yielded a RMSD of 3.0 Å (comparing 81 Cα atoms) (Fig. 4*C*). The general fold of the PARP4 BRCT resembles the structure of PARP1 BRCT domain, though the comparison shows some key differences in the positioning of residues that are important for PARP1 BRCT interaction with nucleic acid. The critical residues for PARP1 BRCT DNA binding are lysines that interact with the DNA phosphate backbone, but also an interaction motif composed of residues Thr420-Gly421-Thr422 motif (22). We identified PARP4 lysine residues K23, K24, K31, and K36 as potentially important due to their vicinity to lysine residues important for PARP1 BRCT binding to DNA (Fig. 4*C*). The Thr420-Gly421-Thr422 motif in PARP1 is Ser38-Phe39-Ser40 in PARP4 and thus not highly conserved. However, the location of Phe39 on the potential nucleic acid–binding surface was intriguing so we also considered Phe39 as a possibly important residue (Fig. 4*C*). Conservation analysis using ConSurf indicated that Phe39 is conserved in 77% of 150 PARP4 homologs (23, 24, 25, 26, 27, 28). We also compared the PARP4 BRCT to the structure the BRCT domain from DNA polymerase μ (29) (Fig. 4*D*; PDB:2HTF), which exhibits dsDNA binding activity (30). The structures superimposed with an RMSD of 4.3 Å (comparing 74 Cα atoms) and indicated that K23 and K31 in PARP4 approximate the position of DNA polymerase μ residues that are crucial for DNA binding (Fig. 4*D*). We narrowed our selection of potential residues to K23, K24, K31, and F39.

### PARP4 BRCT mutants

We created four mutants of the BRCT domain: K23/24Q, K31Q, F39Q, and F39A. We mutated lysine residues to glutamines to remove the positive charge that might interact with the negative charge on RNA/DNA, while likely maintaining the local structure by keeping a polar residue in place. A double mutant to glutamine was created for the two neighboring lysine residues (K23/24Q), thus removing both charges and potentially limiting any redundancy or compensation at this location that might have occurred with single mutants. We mutated Phe39 to glutamine and to alanine to test the importance of this region.

**Figure 5:**
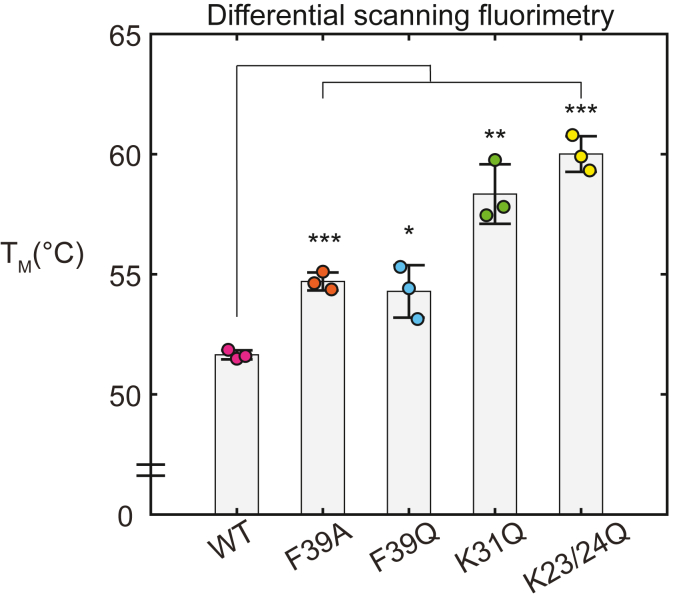
**Relative thermal stability of BRCT domain constructs.** Differential scanning fluorimetry analysis. The experiments were performed with 5 μM PARP4 BRCT domain (WT, F39A, F39Q, K31Q, or K23/24Q). The individual measurements from independent biological replicates are plotted. The bars represent the averages and error bars represent SDs. The apparent *T*_M_ of WT BRCT domain was 51.6 °C ± 0.2 deg, whereas BRCT mutants yielded the following T_M_ values: F39A, 54.7 °C ± 0.4 deg; F39Q, 54.3 °C ± 1 deg; K31Q, 58 °C ± 1 deg; K23/24Q, 60.0 °C ± 0.7 deg. Two-sample, two-sided *t*-tests were used to compare the *T*_M_ values. ∗ = *p* < 0.05, ∗∗ = *p* < 0.005, and ∗∗∗ = *p* < 0.0005. vtRNA, vault RNA.

We tested whether the mutations had an effect on BRCT domain stability using differential scanning fluorimetry (DSF) to measure a *T*_M_ of each mutant relative to the WT BRCT domain. A decreased T_M_ would be interpreted as a decrease in protein stability, which could come from general destabilization of the overall protein fold. In this case, the mutation would not simply be removing important functional atoms, but would rather have a more global impact that could alter the positioning of multiple residues. DSF experiments showed that none of the mutants appeared to destabilize the BRCT domain as none of them exhibited a decrease in T_M_. In fact, each of the mutants exhibited thermal stability that was higher than WT BRCT (Fig. 5). The T_M_ of WT BRCT was measured as 51.6 °C ± 0.2 deg, whereas T_M_ values of 54.7 °C ± 0.4 deg, 54.3 °C ± 1 deg, 58 °C ± 1 deg, and 60.0 °C ± 0.7 deg were obtained for BRCT mutants F39A, F39Q, K31Q, and K23/24Q, respectively. We pursued structural analysis of each mutant to directly compare their structures with WT BRCT.

### Crystal structures of BRCT mutants

**Figure 6:**
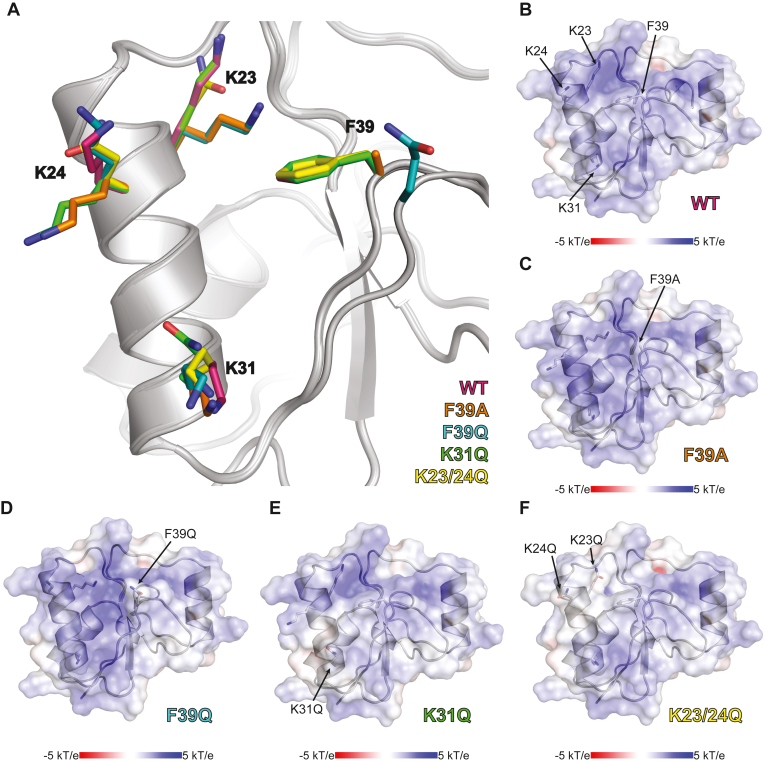
**Structural comparison of PARP4 BRCT domain mutants.***A*, superimposition of the crystal structures of the four mutants of PARP4 BRCT domain. The key residues identified for vtRNA binding are showed in a *stick representation*. Residues from the WT structure are drawn in *pink* (PDB: 9DEV). Residues from mutants F39A and F39Q are, respectively, drawn in *orange* and *teal* (PDB: 9DFR and 9DFQ). Sidechains drawn in *green* are those from the structure of the K31Q mutant (PDB:9DFO). The residues from the structure of the double mutant K23/24Q (PDB: 9DFP) are represented in *yellow*. *B*, electrostatic map of PARP4 BRCT domain (PDB: 9DEV). Potential key residues for nucleic acid binding are labeled. *C–F*, electrostatic map of PARP4 BRCT mutants, where the mutated residues are labeled. vtRNA, vault RNA.

We determined X-ray structures of the four PARP4 BRCT domain mutants. Crystals diffracted between 1.9 and 2.1 Å and belong to space group F23, and the refined structures exhibited respectable geometry and R/R_FREE_ values (Table 2). As expected from thermal stability analysis, the overall BRCT fold was not affected by the different mutations, and superposition of the mutant structures with WT BRCT yielded RMSD values between 0.5 and 0.8 Å (comparing all 763–788 atoms) (Fig. 6*A*). However, there were notable changes in the mutant structures relative to WT that centered around the mutation sites but also propagated to neighboring regions. The perturbations in mutant structures were also viewed in the context of the electrostatic surface potential (Fig. 6, *B*–*F*). Mutants F39A and F39Q both led to a shift in the position of the sidechain of K23, which impacted the charge distribution at the structure surface beyond just the F39 location (Fig. 6, *C* and *D*). The loop that bears residue F39 was repositioned in the F39Q mutant, whereas this loop did not change in the F39A mutant, thus leading to visible changes in the electrostatic surface potential. The K31Q mutant influenced the local surface charge at the K31 position, but also influenced the placement and charge location of the K24 sidechain (Fig. 6, *A* and *E*). In total, BRCT mutants impacted the electropositive surface predicted to bind to nucleic acid without introducing major structural deformations.

### Analysis of BRCT mutants binding to vtRNA

**Figure 7:**
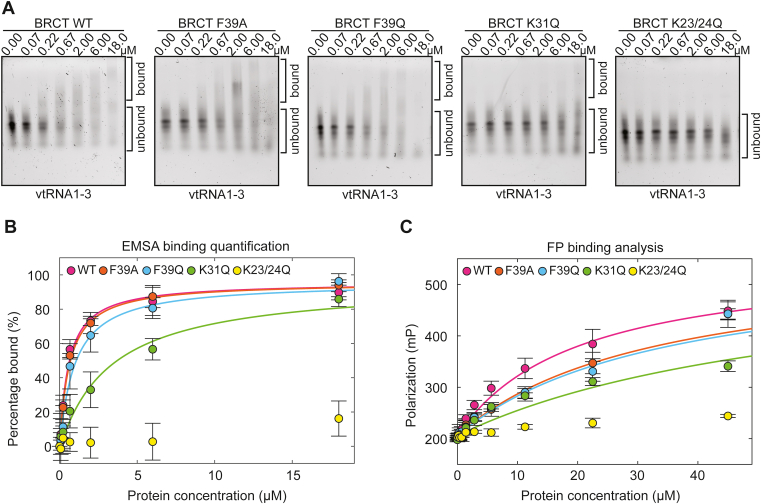
**Binding to vtRNA is impacted by BRCT domain mutations.***A*, EMSA using 20 nM vtRNA1-3 bearing a fluorescent label. PARP4 BRCT domains (WT and mutants) were incubated with vtRNA1-3 at the indicated concentrations. These are representative experiments for each BRCT domain (WT and mutants). Each experiment has been repeated three times for the mutants and four times for the WT. *B*, quantification of the repeats of the EMSA experiments from *panel (A)*. The data points and error bars represent the average and SD of three (or four for WT) independent experiments. A 1:1 binding model was fit to each binding curve. Binding of BRCT WT to vtRNA1-3 yielded an apparent K_D_ of 0.5 ± 0.2 μM. Binding of the BRCT F39A and F39Q domain to vtRNA1-3 yielded apparent K_D_ values of 0.6 ± 0.1 μM and 0.9 ± 0.5 μM, respectively. The K31Q mutant binds vtRNA1-3 with an apparent binding K_D_ of 3 ± 1 μM. BRCT mutant K23/24Q did not show evidence of binding in these conditions. *C*, FP binding assay using 3 nM of vtRNA1-3 bearing a fluorescent label. PARP4 BRCT domains (WT and mutants) were incubated with vtRNA1-3 at the designated concentrations. The plot values represent averages and error bars represent SD of three independent experiments. A 1:1 binding model was fit to the binding curves. Binding of the WT BRCT domain to vtRNA1-3 yielded an apparent K_D_ of 19 ± 4 μM. The F39A and F39Q BRCT mutant bind vtRNA1-3 with apparent K_D_ of 33 ± 2 μM and 35 ± 10 μM, respectively. An apparent K_D_ of 61 ± 12 μM was obtained for the binding of BRCT K31Q mutant to vtRNA1-3. The K23/24Q mutant of PARP4 BRCT domain did not show evidence of binding at the concentrations tested. vtRNA, vault RNA; FP, fluorescence polarization.

EMSA was used to analyze the nucleic acid–binding capacity of the BRCT mutants using vtRNA1-3 as a binding partner. Relative to WT BRCT (0.5 ± 0.2 μM), mutants F39Q and F39A exhibited slightly weakened binding, with estimated K_D_ values of 0.9 ± 0.5 μM and 0.6 ± 0.1 μM, respectively (Fig. 7, *A* and *B* and Table 1 and Fig. S3*C*). The K31Q mutant showed a stronger binding deficiency with an estimated K_D_ value of 3 ± 1 μM. The K23/24Q double mutant of BRCT showed very little to no evidence of binding (Fig. 7, *A* and *B* and Table 1 and Fig. S3*C*). Similar trends in relative affinities were obtained using the FP binding assay (Fig. 7*C* and Table 1 and Fig. S3*D*). Relative to WT BRCT (K_D_ of 19 ± 4 μM), F39Q and F39A showed weakened binding with K_D_ values of 35 ± 10 μM and 33 ± 2 μM, respectively. The K31Q mutant showed a greater deficiency with a K_D_ estimated at 61 ± 12 μM, and the K23/24Q mutant showed no apparent interaction. The mutagenesis thus resulted in four BRCT mutants with intermediate to severe nucleic acid–binding deficiencies and confirms the specificity of the observed BRCT interaction with nucleic acid.

### K23/24Q mutation in the BRCT-WGR-CAT construct

**Figure 8:**
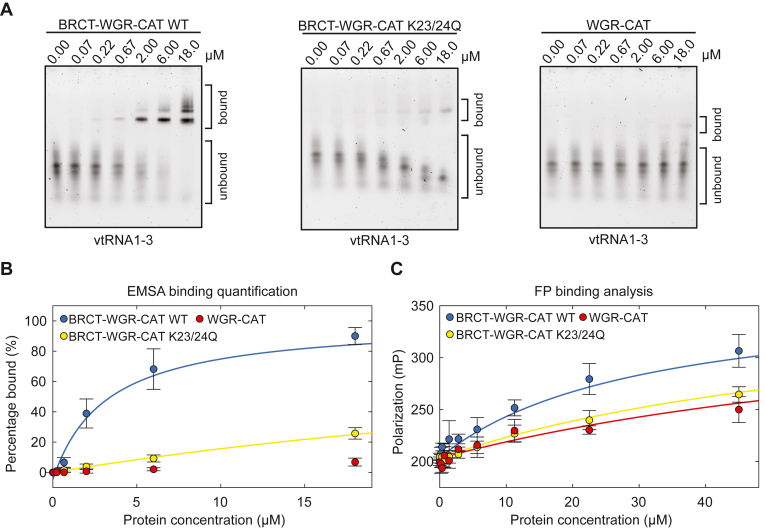
**Analysis of double mutant K23/24Q in BRCT-WGR-CAT.***A*, EMSA using 20 nM vtRNA1-3 bearing a fluorescent label. BRCT-WGR-CAT (WT or K23/24Q) and WGR-CAT fragments were incubated with the nucleic acid at the indicated concentrations. These are representative experiments that have been repeated at least three times each (see Table 1). *B*, quantification of the independent biological repeats of the EMSA experiments from *panel (C)*. The data points and error bars represent the average and SD of three (or six for WT) independent experiments. A 1:1 binding model was fit to each binding curve. Binding of the BRCT-WGR-CAT WT fragment to vtRNA1-3 yielded an apparent K_D_ value of 3 ± 1 μM. A K_D_ of 54 ± 9 μM was estimated for the binding of the BRCT-WGR-CAT K23/24Q fragment to vtRNA1-3. It was not possible to determine a K_D_ value for the binding of the WGR-CAT fragment to vtRNA1-3 in these conditions. *C*, FP binding assay using 3 nM of vtRNA1-3 bearing a fluorescent label. PARP4 WGR-CAT and BRCT-WGR-CAT (WT and K23/24Q) were incubated with vtRNA1-3 at the different indicated concentrations. The plot values represent averages and error bars represent SDs of three independent experiments for the WGR-CAT and BRCT-WGR-CAT K23/24Q (four experiment for WT). A 1:1 binding model was fit to the binding curves. Binding of the WT BRCT-WGR-CAT fragment to vtRNA1-3 yielded an apparent K_D_ of 27 ± 9 μM. BRCT-WGR-CAT K23/24Q and WGR-CAT, respectively, yielded estimated K_D_ values of 71 ± 26 μM and 93 ± 23 μM. CAT, catalytic; vtRNA, vault RNA; FP, fluorescence polarization.

We analyzed the mutant with the strongest phenotype, K23/24Q, in the context of the BRCT-WGR-CAT fragment of PARP4. Using EMSA analysis, we observed a clear defect in binding to vtRNA1-3 for K23/24Q BRCT-WGR-CAT relative to WT (Fig. 8*A*). Whereas WT BRCT-WGR-CAT had an apparent K_D_ of 3 ± 1 μM, the K23/24Q mutant was estimated have a K_D_ of 54 ± 9 μM (Fig. 8*B* and Table 1 and Fig. S4*A*), although this is a rough estimate due to the severely weakened binding. We also used an EMSA experiment to determine the capacity of the WGR-CAT fragment to bind to vtRNA1-3 (Fig. 8*A*). Deletion of the BRCT domain to form the WGR-CAT fragment disrupted vtRNA1-3 binding to such an extent that a reliable K_D_ value could not be estimated in these conditions (Fig. 8*B* and Table 1 and Fig. S4*A*). Using the FP binding assay, we assessed the nucleic binding activity of WT and K23/24Q BRCT-WGR-CAT using vtRNA1-3. The BRCT-WGR-CAT fragment yielded an apparent K_D_ of 27 ± 9 μM in the tested conditions (Fig. 8*C* and Table 1 and Fig. S4*B*). The K23/24 mutant showed clear evidence of a strong deficiency in binding capacity (estimated K_D_ of 71 ± 26 μM), similar to the WGR-CAT fragment that represents a complete BRCT domain deletion (estimated K_D_ of 93 ± 23 μM) (Fig. 8*C* and Table 1 and Fig. S4*B*). Our published study indicated no binding of the WGR-CAT fragment to vtRNA1-2 in a different set of conditions (12). Results from the present study show that the WGR-CAT fragment can in fact interact with vtRNA1-3 under the tested conditions, albeit at a very low level. The K23/24Q mutation of the BRCT-WGR-CAT fragment led to a strong deficiency in the vtRNA1-1 binding capacity of the fragment. Thus, the K23/24Q mutation recapitulated the effect of BRCT domain deletion.

## Discussion

This study focused on the structure of the PARP4 BRCT domain and its interaction with vtRNA. In a previous study, we demonstrated that the BRCT-WGR-CAT fragment binds to vtRNA, and deletion of the BRCT from this fragment (*i.e.* the WGR-CAT) largely disrupted binding (12). It remained possible that BRCT required WGR and/or CAT and functioned together to mediate the interaction; however, we show here that the isolated BRCT domain binds to vtRNA. The BRCT domain alone binds to vtRNA with slightly higher affinity than BRCT-WGR-CAT, suggesting that there might be some level of interaction between BRCT and WGR-CAT that perturbs binding to vtRNA. This observation is reminiscent of the finding that the MARylation activity of BRCT-WGR-CAT can be increased by deletion of the BRCT domain (12). Further structural studies will be required to understand potential interactions between the domains of BRCT-WGR-CAT of PARP4.

The results indicate the human PARP4 BRCT can be classified as a nucleic acid–binding module. The structure is comparable to other nucleic acid–binding BRCT domains like the ones found in PARP1 and DNA polymerase μ. Moreover, the residues that we targeted for mutagenesis to disrupt nucleic acid binding are in similar locations as mutants that have been used to disrupt the DNA binding activity of BRCT domains in PARP1 and DNA polymerase μ. The actual cellular target of the nucleic acid–binding activity of PARP4 BRCT domain is yet to be defined and could vary depending on cellular localization. We have focused on interaction with vtRNA due to the colocalization of PARP4 and vtRNA to the interior of the vault particle. The BRCT exhibited similar binding to all vtRNAs, and it also bound with comparable affinity to a nucleic acid structure in which the vtRNA1-3 ribonucleotides were converted to deoxynucleotides, vtDNA1-3. Binding analysis with BRCT-WGR-CAT furthermore showed that there is no preferential binding to a specific region of vtRNA1-3. This could reflect that the BRCT domain is largely recognizing duplex regions without a strong selection of DNA *versus* RNA nucleotides and could also indicate a general nucleic acid–binding function for the PARP4 BRCT domain that could serve multiple cellular roles. It remains possible that the PARP4 BRCT domain could also interact with a different or additional electronegative binding partner *in vivo*. Further investigations are needed to determine other potential interacting partners.

Our mutagenesis results led to PARP4 BRCT variants that exhibited a range of deficiencies in binding to vtRNA, with the K23/24Q double mutant resulting in complete loss of BRCT binding to vtRNA. The mutations resulted in perturbations to electropositive regions of the BRCT surface that are expected to engage nucleic acid. The mutations maintained the integrity of the BRCT domain as evidenced by measurement of relative thermal stability. Indeed, the mutations tended to increase the thermal stability relative to WT BRCT. This observation could potentially be explained by changes in the polar surface area that impact thermostability (31). The double mutant K23/24Q led to the most notable increase in thermal stability. We noted an additional water molecule adjacent to residue 23 when it was mutated to glutamine in the K23/24Q structure. This local increase in hydrogen bonding potential conceivably contributed to the increase in thermal stability of this BRCT variant, as an increase in surface hydrogen bonding is a common explanation for enhanced thermostability (31). The K23/24Q mutation was also effective at considerably reducing vtRNA interaction in the BRCT-WGR-CAT fragment, and the deficiency was comparable to that of a complete BRCT deletion. The mutagenesis results and structural analysis support the conclusion that human PARP4 BRCT specifically engages nucleic acid.

More structural and biochemical investigation are needed to fully understand the regulation and structure of human PARP4, in particular the manner in which the multiple domains function together. These types of insights will aid investigation of the biological roles of PARP4. The BRCT mutant K23/24Q should serve as a valuable tool to investigate the nucleic acid–binding cellular functions of PARP4.

## Experimental procedures

### Expression constructs and mutagenesis

Human PARP4 residues 1 to 573 (*i.e.* BRCT-WGR-CAT fragment) were expressed from a pET28 expression vector with an N-terminal his-tag and SUMO-tag (gift from Dr Michael Cohen). Using site-directed mutagenesis using the QuickChange protocol (Agilent), a stop codon was introduced in this construct to yield the BRCT fragment (residues 1–100). BRCT mutants F39A, F39Q, K31Q, and K23/24Q were obtained using the BRCT domain plasmid and QuickChange mutagenesis. The BRCT domain was deleted from the BRCT-WGR-CAT fragment using QuickChange to yield the WGR-CAT construct (residues 140–573). The K23/24Q mutant of BRCT-WGR-CAT was created using QuickChange. All plasmids were sequence verified using Oxford Nanopore Technology (Plasmidsaurus) or automated Sanger sequencing.

### Protein expression and purification

PARP4 BRCT WT, F39A, F39Q, K31Q, and K23/24Q were expressed in *Escherichia coli* strain Rosetta2(DE3) (Novagen). The cells were grown at 37 °C in LB medium until the absorbance at 600 nm reached ∼0.6, and then induced with 200 μm IPTG at 16 °C for 20 h. The cells were resuspended in 20 mM Hepes, pH 8.0, 500 mM NaCl, 0.5 mM Tris[2-carboxyethyl] phosphine (TCEP), 0.1% Nonidet P-40 (NP40), 1 mM PMSF, protease inhibitors (antipain, pepstatin, leupeptin, benzamidine, and aprotinin) and then lysed using a cell disrupter (Avestin). The lysate was then centrifugated for 2 h at 40,000 g. The supernatant was filtered then loaded onto a 5-ml HP chelating column (Cytiva) charged with Ni(II) and pre-equilibrated with equilibration buffer containing 20 mM Hepes, pH 8.0, 0.5 mM TCEP, 500 mM NaCl, 1 mM PMSF, protease inhibitors, and 10% glycerol. The column was washed with 10 column volumes of 20 mM Hepes, pH 8.0, 500 mM NaCl, 0.5 mM TCEP, 1 mM PMSF, protease inhibitors, 10% glycerol, and 20 mM imidazole. The column was then washed with 10 column volumes of the same buffer supplemented to 1 M NaCl and then again with 10 column volumes of 500 mM NaCl buffer. The proteins were then eluted with 4 column volumes of buffer supplemented with 400 mM imidazole. The N-terminal tag was cut using TEV protease during overnight dialysis at 4 °C into 20 mM Hepes, pH 8.0, 150 mM NaCl, 0.5 mM TCEP, and 10% glycerol. The protein mixture was then passed over a second Ni(II)-affinity column, with the untagged protein in the flow-through and the tag remaining on the column. The flow-through protein was then passed over a size-exclusion chromatography column mounted on a chromatography system (AKTA purifier) and maintained in 20 mM Hepes pH 8.0, 150 mM NaCl, 0.1 mM TCEP, and 0.1 mM EDTA. A HiLoad 16/600 Superdex75 column (Cytiva) was used for gel filtration. Aliquots of concentrated proteins were flash-frozen in liquid nitrogen and stored at −80 °C. PARP4 BRCT-WGR-CAT (WT and K23/24Q) and WGR-CAT were expressed and purified as described above, with the exception that a Hiprep 26/60 Sephacryl S200 column (Cytiva) was used for gel filtration, and an additional chromatographic step was performed after the second Ni(II) affinity column and prior to gel filtration. The flow-through of the second Ni(II) affinity was slowly supplemented with ammonium sulfate to a final concentration of 1 M and then loaded on a 5-ml HiTrap Butyl HP column (Cytiva) equilibrated with 50 mM sodium phosphate pH 7 and 1 M ammonium sulfate. After washing, the column was eluted over a 40-column volume gradient to 50 mM sodium phosphate pH 7.

### Nucleic acids

The vtRNAs (vtRNA1-1: NCBI accession number NR_026703, vtRNA1-2: NCBI accession number NR_026704, and vtRNA1-3: NCBI accession number NR_026705) were chemically synthesized by Integrated DNA Technologies bearing a 5′-6-FAM fluorescent group. vtRNA1-3 sequence was converted to DNA and synthesized with a 5′-6-FAM fluorescent group. The sequences for the vtRNA1-3 fragments are shown in Table S1. The nucleic acids were annealed at 10 μM in an annealing buffer containing 5 mM MES pH 6.5, 25 mM NaCl, and 5 mM MgCl_2_. The annealing procedure consisted of heating the samples to 60 °C and then slowly lowering the temperature to 20 °C over a period of 1 h in a thermocycler.

### FP binding assay

PARP4 BRCT-WGR-CAT (WT and K23/24Q), WGR-CAT, and BRCT (WT, F39Q, F39A, K31Q, and K23/24Q) were incubated for 30 min at room temperature with fluorescently labeled nucleic acid (vtRNA1-1, vtRNA1-2, vtRNA1-3, vtDNA1-3, or vtRNA1-3 fragments). For the comparison of vtRNA1-1, vtRNA1-2, and vtRNA1-3 binding to BRCT and BRCT mutants, we used the lowest concentration of fluorescent vtRNA (3 nM) that provided a reasonable signal, due to a limited amount of these specialty vtRNA reagents. To verify that the 3 nM concentration would not pose a limit on the reliability of our measurements due to low signal, we compared the binding of PARP4 BRCT domain to vtRNA1-3 using either 3 nM or 30 nM fluorescent probe and obtained similar K_D_ values (Fig. S5). For the experiments comparing the binding of the BRCT-WGR-CAT to vtRNA1-3 and fragments thereof (probes 1–4), we used 15 nM nucleic acid. The FP binding assay was performed in a buffer containing 50 mM Tris pH 7.5, 0.05% NP40, 2 mM DTT, 50 mM NaCl, 5% glycerol, 1 mM MgCl_2_, and 25 μg/ml bovine serum albumin. A VictorV plate reader (PerkinElmer) was used to measure FP. A 1:1 binding model was fit to the data in MATLAB (MathWorks). For mutants with weakened binding that did not exhibit a clear binding plateau, the refined plateau value in the fitted curve was constrained by a set of curves that were cofitted and included WT protein with a clearer binding plateau. Experiments with the BRCT WT fragment with vtRNA1-3 were repeated nine times and ten times for the BRCT-WGR-CAT fragment with vtRNA1-3, whereas all other experiments were repeated three times. All experiments were performed as independent biological replicates.

### Electrophoretic mobility shift assay

PARP4 BRCT-WGR-CAT (WT and K23/24Q), WRG-CAT, and BRCT (WT, F39A, F39Q, K31Q, and K23/24Q) (0–36 μM) were prepared on ice in 2X binding buffer (100 mM Tris pH 7.5, 100 mM NaCl, and 8% Ficoll PM400). Annealed nucleic acids (40 nM) were prepared on ice in 2X RNA buffer (0.1% NP40 and 4 mM DTT). Equal parts of PARP4 and nucleic acid were mixed, yielding final concentrations of 20 nM nucleic acid, 50 mM Tris pH 7.5, 50 mM NaCl, 4% Ficoll PM400, 0.05% NP40, 2mM DTT, and PARP4 between 0 and 18 μM. The samples were incubated for 30 min at room temperature. Mini-PROTEAN TGX Stain-Free gels (Bio-Rad; 7.5%) were pre-run in Tris-glycine buffer at 4 °C (100 V, 1 h), and then the loaded samples were resolved at 60 V for 1 h at 4 °C. Experiments performed with vtRNA1-3 were visualized by staining with SYBR Gold 1X in Tris-glycine buffer for 30 min. Experiments performed with the fragments of vtRNA1-3 (probes 1–4) were visualized by fluorescence detection. All images were acquired on a ChemiDoc MP (Bio-Rad). The intensity of imaged gel bands was quantified using ImageJ (NIH; https://imagej.net). For the experiments using the BRCT domain (WT, F39A, F39Q, K31Q, and K23/24Q) that led to a diffuse shifted band, we analyzed the amount of unbound probe at each protein concentration relative to the no protein condition, yielding a fraction unbound that was converted to percentage bound: 100 × (1 – fraction unbound). For the experiments performed with BRCT-WGR-CAT (WT and K23/24Q) and WGR-CAT, the percentage of nucleic acid bound was calculated as the ratio of the shifted band intensity over the total nucleic acid intensity (bound + unbound). A 1:1 binding model was fit to the data in MATLAB (MathWorks). For mutants with weakened binding that did not exhibit a clear binding plateau, the refined plateau value in the fitted curve was constrained by a set of curves that were cofitted and included WT protein with a clear binding plateau. Three independent experiments were performed for each binding curve, except for BRCT WT with vtRNA1-3 that was repeated seven times and BRCT-WGR-CAT with vtRNA1-3 that was repeated 12 times. All experiments were performed as independent biological replicates.

### Crystallization and structure determination

PARP4 BRCT domain WT was crystallized using sitting drop vapor diffusion at room temperature in 2 M ammonium sulfate and 0.15 M citric acid pH 3.5. Mutant F39Q was crystallized using sitting drop vapor diffusion at room temperature in 2 M ammonium sulfate and 0.1 M sodium acetate pH 4.6. Mutants F39A, K31Q, and K23/24Q were crystallized using sitting drop vapor diffusion in PEG3350 (23–31%), 0.2 M ammonium sulfate, and 0.1 M sodium acetate pH 4.6. Crystals were obtained with protein concentrations between 6 and 10 mg/ml. Crystals of the WT BRCT were cryo-protected by a solution composed of the crystallization condition supplemented with 20% glycerol. The cryo-protection solution for BRCT F39Q and F39A crystals was composed of the crystallization solution supplemented with sucrose (15–20%). Crystals from BRCT mutants K31Q and K23/24Q were cryo-protected by a solution composed of the crystallization solution supplemented with 15% ethylene glycol and 20% glycerol, respectively. The crystallization solution of each crystal was exchanged with the cryo-protection solution in the sitting drop before flash-cooling with liquid nitrogen.

X-ray diffraction data for PARP4 BRCT WT was collected at beamline 8.3.1 of the Advance Light Source. X-ray diffraction data for PARP4 BRCT F39A, F39Q, K31Q, and K23/24Q were collected on the Canadian Macromolecular Crystallography Facility bending magnet beamline of the Canadian Light Source. All diffraction data were processed using X-ray Detector Software (https://xds.mr.mpg.de) (32) and AIMLESS in the CCP4 suite (Collaborative Computing Project, No. 4; https://www.ccp4.ac.uk) (33, 34) (Table 2). The structures were determined by molecular replacement using PHASER-MR (35) in the PHENIX suite (https://www.phenix-online.org) (36). The AlphaFold predicted structure of PARP4 BRCT domain (residues 1–100) was used as a search model for BRCT WT diffraction data (20, 37). The search model was manually adjusted using COOT (38), and the structure was refined using REFMAC5 (39, 40, 41, 42). The refined X-ray structure of BRCT WT was used as a starting model for BRCT mutants (F39Q, F39A, K31Q, and K23/24Q). Adjustments to these models were made in COOT (38), and the structures were refined using REFMAC5 (39, 40, 41, 42).

### Differential scanning fluorimetry

DSF experiments were performed using 5 μM protein and 5X Sypro Orange (Invitrogen) in a final volume of 20 μl. Fluorescence emission was measured while the temperature was increased from 20 to 85 °C (0.02 °C/s with 25 acquisitions per degree) in a Roche Light-Cycler 480 RT-PCR. Experiments were performed in the following buffer: 25 mM Hepes pH 8.0, 150 mM NaCl, 1 mM EDTA, and 0.1 mM TCEP. A Boltzmann sigmoid was fit to the data to determine the apparent *T*_M_ values using MATLAB (MathWorks). All experiments were performed as independent biological replicates.

## Data availability

The atomic coordinates and structure factors have been deposited in the Protein Data Bank (https://www.rcsb.org) and are publicly available as of the date of publication. Accession codes are PDB: 9DEV (PARP4 BRCT WT), PDB: 9DFR (PARP4 BRCT F39A), PDB: 9DFQ (PARP4 BRCT F39Q), PDB: 9DFO (PARP4 BRCT K31Q), and PDB: 9DFP (PARP4 BRCT K23/24Q). Any additional information required to reanalyze the data reported in this article is available from the corresponding author upon request.

## Supporting information

This article contains supporting information (44).

## Conflict of interest

The authors declare that they have no conflicts of interest with the contents of this article.
