# Supplementary material for: Crystal structure and mutagenesis of a nucleic acid–binding BRCT domain in human PARP4
Source: J Biol Chem. 2025 May 22;301(6):110277. doi: 10.1016/j.jbc.2025.110277 (PMC12212138; doi:10.1016/j.jbc.2025.110277)
Supplement: Supporting Information [file mmc1.pdf]

Supporting information

Crystal structure and mutagenesis of a nucleic acid-binding BRCT domain in human PARP4

Léonie Frigon and John M. Pascal

Table of Contents		Page
**Figure S1**	Predicted secondary structure of vault RNAs	S-2
**Figure S2**	Replotting of the binding analysis from Figures 1 and 2	S-3
**Figure S3**	Replotting of the binding analysis from Figures 3 and 7	S-4
**Figure S4**	Replotting of the binding analysis from Figure 8	S-5
**Figure S5**	Comparison of PARP4 BRCT domain binding to vtRNA1-3 using two different probe concentrations in the FP assay	S-6
**Table S1**	Nucleic acid sequences	S-7

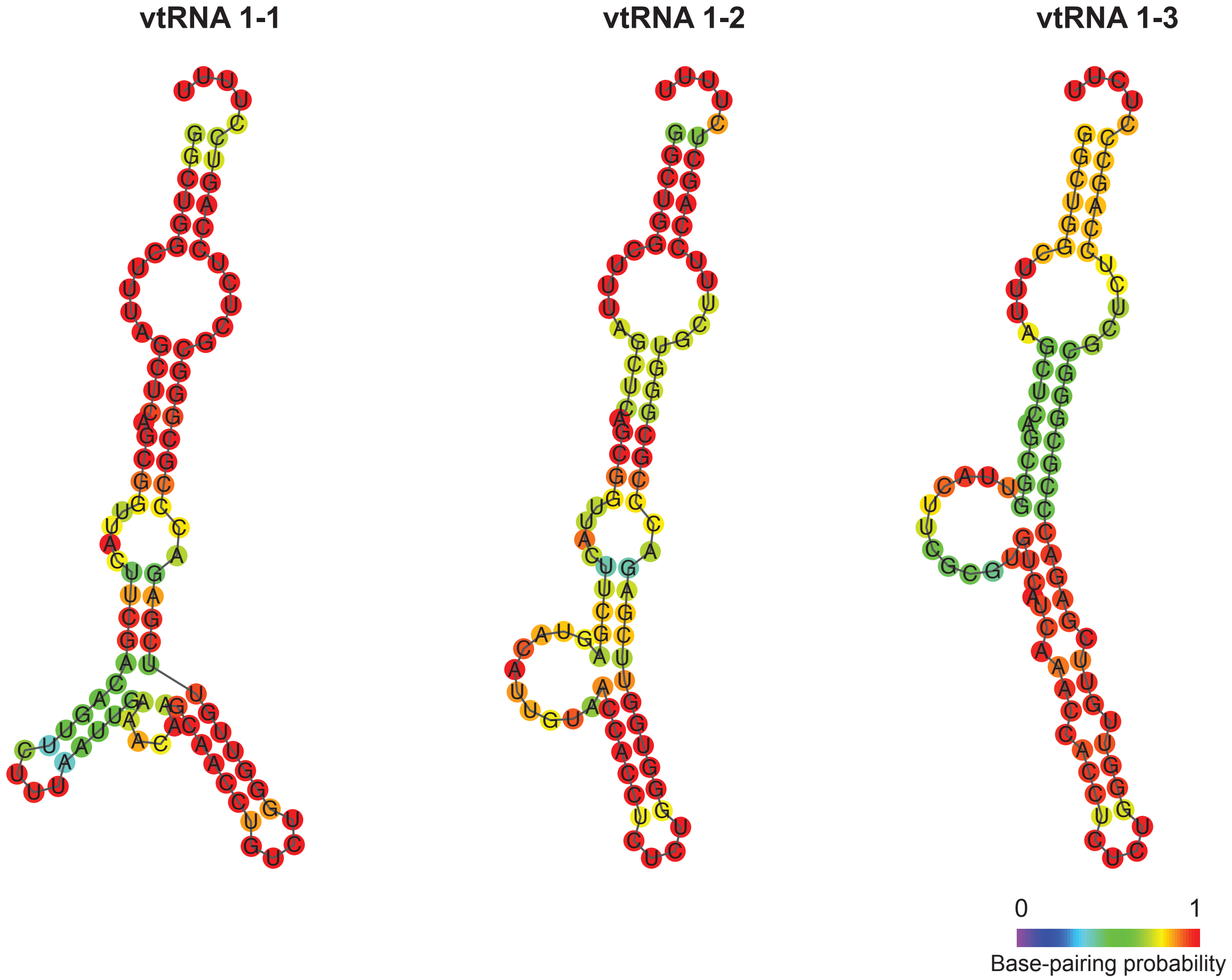

Figure S1. Predicted secondary structures of vault RNAs.
Predictions of vault RNA secondary structures using RNA fold (44). Base-pairing probabilities are visualized using the indicated color scale from 0 to 1, with 1 in red indicating that the prediction of base-pairing (or not base pairing) is high probability.

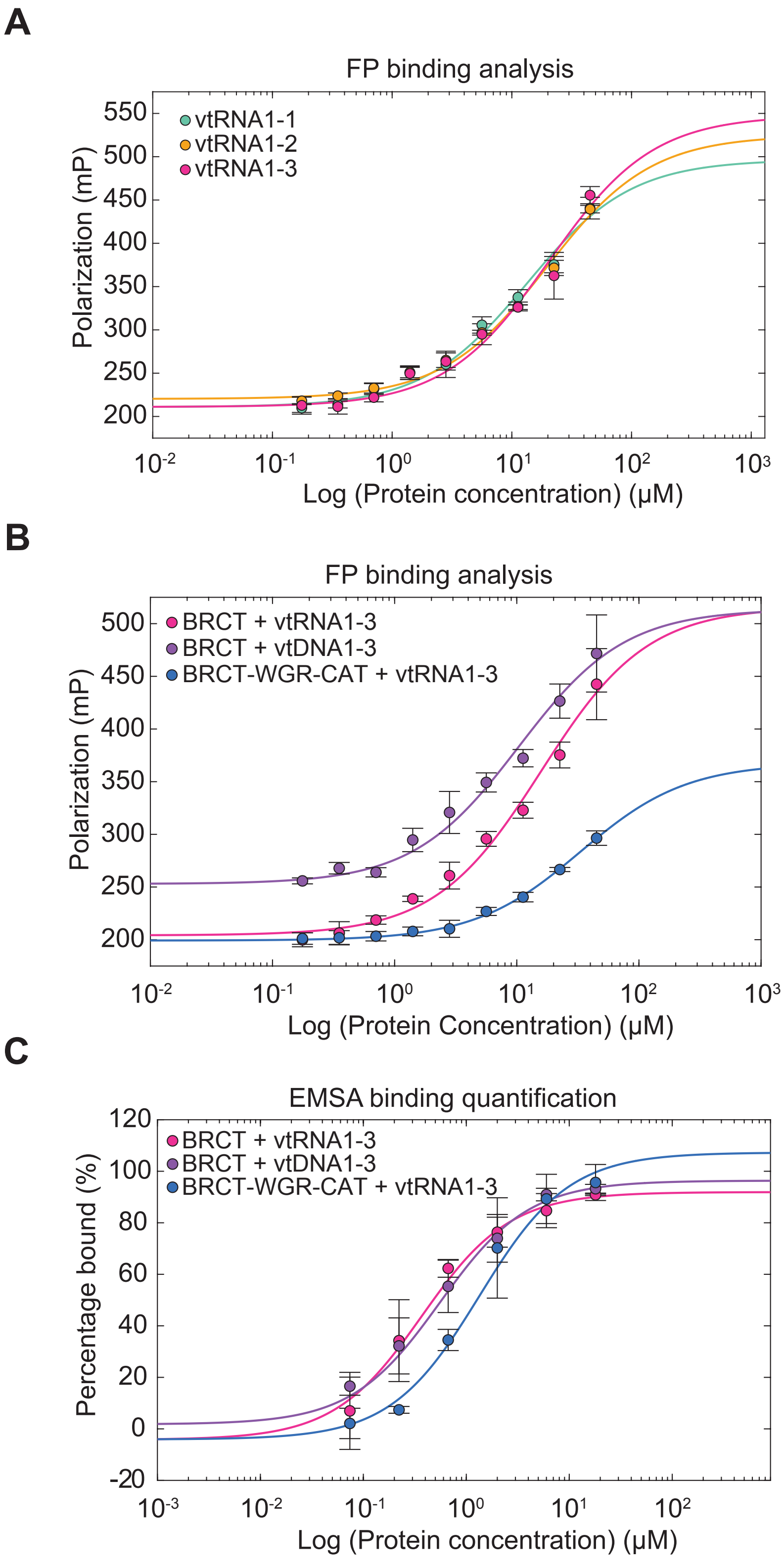

Figure S2. Replotting of the binding analysis from Figures 1 and 2.
A. Replotting of FP binding experiment with vtRNAs and PARP4 BRCT from Figure 1 using Log (Protein concentration) vs Polarization (mP). **B.** Replotting of FP binding experiment with vtRNA1-3 and vtDNA1-3 from Figure 2 as Log (Protein concentration) vs Polarization (mP). **C.** Replotting of EMSA binding experiment with vtRNA1-3 and vtDNA1-3 from Figure 2 as Log (Protein concentration) vs Percentage bound (%).

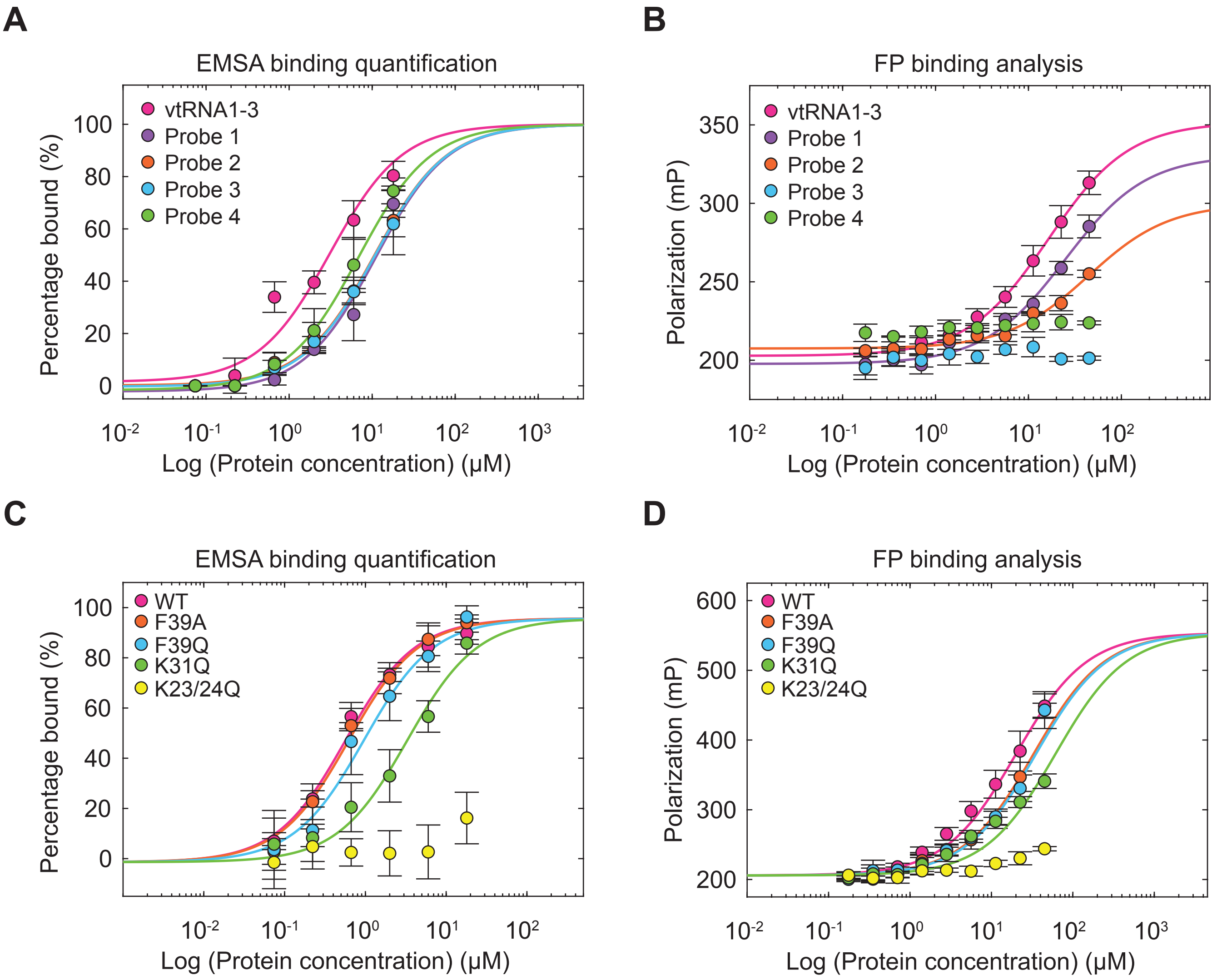

Figure S3. Replotting of the binding analysis from Figures 3 and 7.

A. EMSA binding experiment of PARP4 BRCT-WGR-CAT with vtRNA1-3 fragments from Figure 3 replotted as Log (Protein concentration) vs Percentage bound (%). **B.** FP binding experiment of PARP4 BRCT-WGR-CAT with vtRNA1-3 fragments from Figure 3 replotted as Log (Protein concentration) vs Polarization (mP). **C.** Replotting of EMSA binding experiment with BRCT mutants binding to vtRNA1-3 from Figure 7 as Log (Protein concentration) vs Percentage bound (%). **D.** Replotting of FP binding experiment with BRCT mutants binding to vtRNA1-3 from Figure 7 as Log (Protein concentration) vs Polarization (mP).

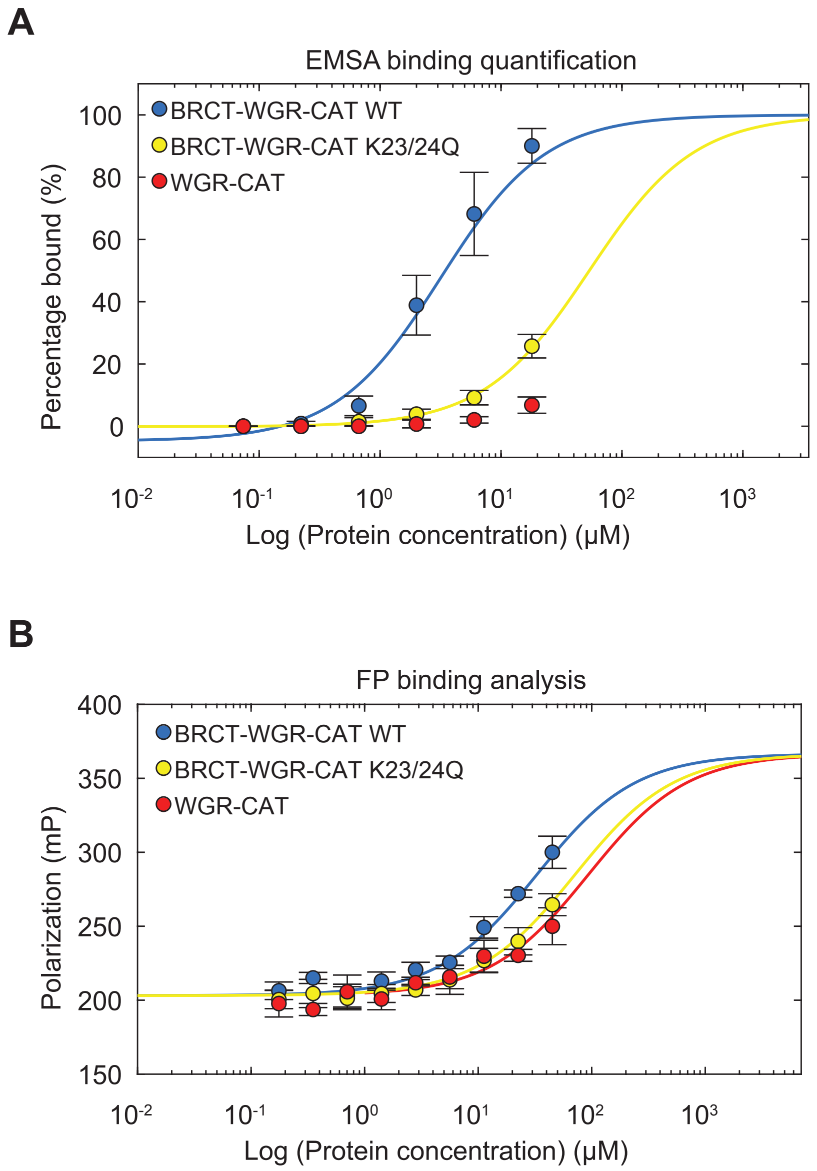

Figure S4. Replotting of the binding analysis from Figure 8.
A. Replotting of EMSA binding experiment with BRCT-WGR-CAT WT and K23/24Q with vtRNA1-3 from Figure 8 as Log (Protein concentration) vs Percentage bound (%). **B.** Replotting of FP binding experiment with BRCT-WGR-CAT WT and K23/24Q with vtRNA1-3 from Figure 8 as Log (Protein concentration) vs Polarization (mP).

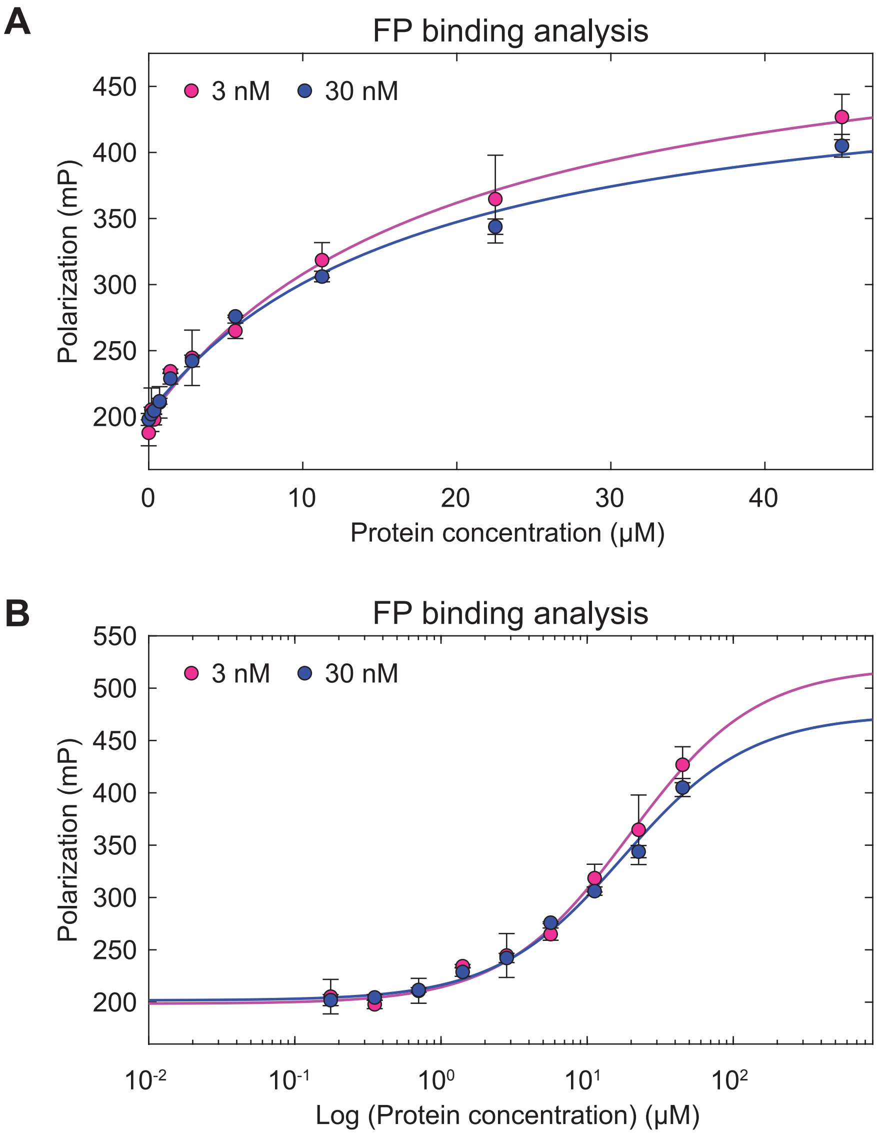

Figure S5. Comparison of PARP4 BRCT domain binding to vtRNA1-3 using two different probe concentrations in the fluorescence polarization (FP) assay.
A. FP binding assay using 3 or 30 nM vtRNA1-3 bearing a fluorescent label. The BRCT domain was incubated with the nucleic acid at the designated concentrations. The data points and error bars represent the average and standard deviation of the independent experiments. A 1:1 binding model was fit to the binding curves, yielding an apparent K_D of 19 ± 3 μM for the experiment with 3 nM probe, and a K_D value of 18 ± 3 μM using 30 nM probe. **B.** Same experiment as in panel A but with the x-axis plotted as Log (Protein concentration). The 3 nM and 30 nM experiments yielded a similar K_D value for the binding of PARP4 BRCT to vtRNA1-3, indicating that the lower concentration of nucleic acid yields reliable results.

Table S1. Nucleic acid sequences

Nucleic acid	Sequence	Predicted secondary structure
vtRNA1-1	5'-6-FAM-<br>GGCUGGCUUUAGCUCAGCGGUUACUUCGACA<br>GUUCUUUAAUUGAAACAAGCAACCUGUCUGG<br>GUUGUUCGAGACCCGCGGGCGCUCUCCAGU<br>CCUUUU-3'	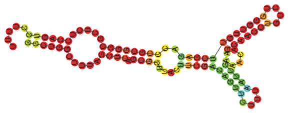
vtRNA1-2	5'-6-FAM-<br>GGCUGGCUUUAGCUCAGCGGUUACUUCGAGU<br>ACAUUGUAACCACCUCUCUGGGUGGUUCGAG<br>ACCCGCGGGUGCUUUCCAGCUCUUUU-3'	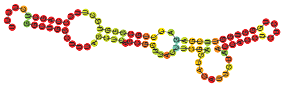
vtRNA1-3	5'-6-FAM-<br>GGCUGGCUUUAGCUCAGCGGUUACUUCGCG<br>UGUCAUCAAACCACCUCUCUGGGUUGUUCGA<br>GACCCGCGGGCGCUCUCCAGCCCUCUU-3'	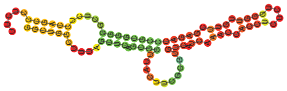
vtDNA1-3	5'-6-FAM-<br>GGCTGGCTTTAGCTCAGCGGTTACTTCGCGTG<br>TCATCAAACCACCTCTCTGGGTTGTTCGAGACC<br>CGCGGGCGCTCTCCAGCCCTCTT-3'	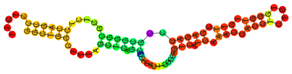
Probe 1	5'-6-FAM-<br>GGCUGGCUUUAGCUCAGCGG-3'<br><br>5'-CCGCGGGCGCUCUCCAGCCCUCUU-3'	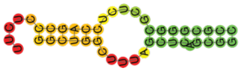
Probe 2	5'-6-FAM-<br>GUCAUCAAACCACCUCUCUGGGUUGUUCGAG<br>AC-3'	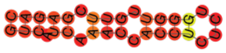
Probe 3	5'-6-FAM-<br>CCGCGGGC-3'<br><br>5'-GCUCAGCGG-3'	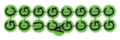
Probe 4	5'-6-FAM-<br>CCGCGGGC-3'<br><br>5'-GCUCGCGG-3'	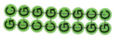
